# Balancing Precision and Risk: Should Multiple Detection Methods Be Analyzed Separately in N-Mixture Models?

**DOI:** 10.1371/journal.pone.0049410

**Published:** 2012-12-12

**Authors:** Tabitha A. Graves, J. Andrew Royle, Katherine C. Kendall, Paul Beier, Jeffrey B. Stetz, Amy C. Macleod

**Affiliations:** 1 School of Forestry, Northern Arizona University, Flagstaff, Arizona, United States of America; 2 United States Geological Survey, Patuxent Wildlife Research Center, Laurel, Maryland, United States of America; 3 United States Geological Survey, Northern Rocky Mountain Science Center, Glacier Field Station, Glacier National Park, Montana, United States of America; 4 Montana Cooperative Wildlife Research Unit, University of Montana, Missoula, Montana, United States of America; 5 University of Montana Cooperative Ecosystem Studies Unit, Glacier Field Station, Glacier National Park, Montana, United States of America; Sapienza University of Rome, Italy

## Abstract

Using multiple detection methods can increase the number, kind, and distribution of individuals sampled, which may increase accuracy and precision and reduce cost of population abundance estimates. However, when variables influencing abundance are of interest, if individuals detected via different methods are influenced by the landscape differently, separate analysis of multiple detection methods may be more appropriate. We evaluated the effects of combining two detection methods on the identification of variables important to local abundance using detections of grizzly bears with hair traps (systematic) and bear rubs (opportunistic). We used hierarchical abundance models (N-mixture models) with separate model components for each detection method. If both methods sample the same population, the use of either data set alone should (1) lead to the selection of the same variables as important and (2) provide similar estimates of relative local abundance. We hypothesized that the inclusion of 2 detection methods versus either method alone should (3) yield more support for variables identified in single method analyses (i.e. fewer variables and models with greater weight), and (4) improve precision of covariate estimates for variables selected in both separate and combined analyses because sample size is larger. As expected, joint analysis of both methods increased precision as well as certainty in variable and model selection. However, the single-method analyses identified different variables and the resulting predicted abundances had different spatial distributions. We recommend comparing single-method and jointly modeled results to identify the presence of individual heterogeneity between detection methods in N-mixture models, along with consideration of detection probabilities, correlations among variables, and tolerance to risk of failing to identify variables important to a subset of the population. The benefits of increased precision should be weighed against those risks. The analysis framework presented here will be useful for other species exhibiting heterogeneity by detection method.

## Introduction

Many species exhibit individual heterogeneity in their susceptibility to different detection methods. When estimating population size is the goal, using multiple detection methods can reduce heterogeneity, increase accuracy and precision, and reduce cost through increasing the number, kind, and distribution of individuals sampled [1]–[3], e.g., [4]–[6]. However, we know of no research evaluating the benefits and risks of combining multiple detection methods when the goal is identification of environmental variables influencing local abundance, as in N-mixture models [7]. N-mixture models link a Poisson or negative binomial distribution that represents the local abundance of individuals with a binomial detection process that yields observed counts of individuals. Covariates for the two levels of the hierarchical process permit identification of variables explaining either abundance or detection [8], [9].

Analyzing detection methods jointly may be appropriate and improve precision of estimates when both methods sample the entire population, when biases in sampling different components of the population can be accounted for with detection covariates, or when methods sample different subsets of the population, but both subsets are influenced similarly by environmental covariates. In contrast, analyzing datasets separately may be more appropriate when individuals more susceptible to capture via one method are influenced by the landscape differently than individuals not susceptible to that method. For instance if a bear that has been harassed with rubber bullets avoids hair snags (one detection method that uses a scent lure) and avoids high human use areas (habitat displacement influencing local abundance), combining detections of hair snags with a second detection method may mask the influence of high human use areas.

We test a set of hypotheses and conduct a thought experiment to evaluate whether joint or separate analysis of multiple detection methods is most appropriate in a dataset from a natural population, when truth is unknown. If both methods sample the same population, the use of either data set alone should (1) lead to the selection of the same variables as important and (2) provide similar estimates of relative local abundance. In contrast, if different subsets of the population are sampled with each method and these groups respond differently to the landscape, separate analyses would identify different variables as important. If the variables identified as important differ greatly, the distribution of local abundance should also vary greatly. On the other hand, we hypothesized that the inclusion of 2 detection methods versus either method alone should (3) yield more support for variables identified in both of the single method analyses (i.e. fewer variables and models with greater weight), and (4) improve precision of covariate estimates for variables selected in both separate and combined analyses because sample size is larger.

To evaluate these hypotheses we used a model that includes multiple detection methods in N-mixture models that we developed for the northern quarter of a population of grizzly bears (*Ursus arctos*) sampled in the year 2000 [10]. Previous work [6], [11] found that inclusion of both detection methods increased the number of bears detected, particularly males, which led to increased precision of overall abundance estimates, at relatively low cost. To obtain high precision (coefficient of variation = 3.8%) for a population abundance estimate across the whole ecosystem, the same 2 kinds of data (systematically-located hair trap and opportunistically monitored bear rubs) were collected in 2004 [12]. We demonstrate an assessment of the pros, cons, and appropriateness of analyzing multiple detection methods separately or jointly for the goal of identifying covariates influencing local abundance in a natural population.

## Methods

### Field and Genetic Methods

**Figure 1:**
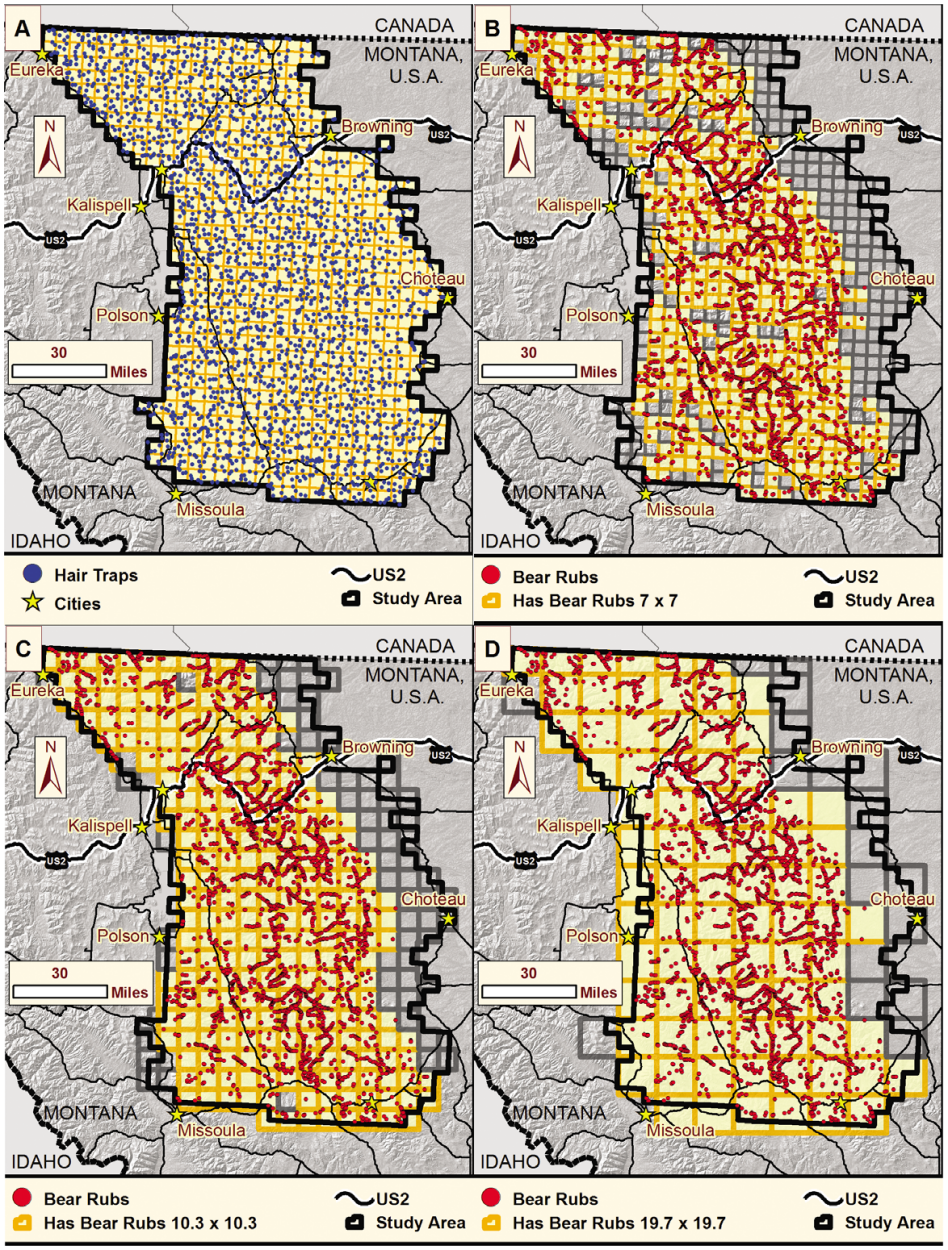
Sampling sites. Location of (A) hair traps distributed on a 7×7 km grid and (B) bear rubs sampled to detect grizzly bears in northwestern Montana, USA, in 2004. Position of the same bear rubs within a (C) 10.3×10.3 km (median female home range size)) and (D) 19.7×19.7 km (median male home range size) grid scale used for analysis. Highlighted cells contain both hair traps and bear rubs and were used in our analyses comparing variable selection with each method.

We used 2 independent, concurrent, non-invasive genetic (hair) sampling methods to detect grizzly bears across a 31,410 km^2^ area that encompassed occupied range associated with the Northern Continental Divide Grizzly Bear Recovery Zone (USFWS 1993) in northwestern Montana. (1) We distributed hair traps using a systematic grid of 641 7×7- km cells during 15 June–18 August 2004 (Fig. 1A). We placed one trap in a different location in each cell during 4 14-day sampling occasions. When bears crossed the barbed-wire corral to approach the scent lure in the center, they often left behind hair. Trap locations within the grid cell were selected *a priori* with consistent criteria based on maps and expert knowledge of bear activity, natural travel routes, seasonal vegetation, and recent wildfire severity. (2) We also collected hair from bear rubs from 15 June–15 September 2004. Bears naturally rub on trees and other objects. We placed strands of barbed wire on 4,795 rubs to facilitate hair collection. We sampled bear rubs along trails, forest roads, and power and fence lines. At each visit to a hair trap or bear rub, we collected all hairs so only newly deposited hairs would be collected at the next visit. Due to paucity of resources, 198 cells of 641 did not have any bear rub sampling effort (Fig. 1B). From hair collected with both methods, we genotyped 545 individuals at 16 microsatellite loci plus a locus identifying sex. Considering the multiple, powerful measures to prevent and correct genotyping errors, it is unlikely that our data included any misidentified individuals [12]. The study area was highly heterogeneous, with multiple land-ownerships, two climatic zones, rugged and flat terrain, and varied land-use. See [12] for further details on study area, sampling design, genetic methods, and ecosystem-wide results.

All necessary permits were obtained for the described field studies (Supporting Information S1).

## Modeling Methods

We conducted analyses for females using a grid of cells representing median female home range size (10.3×10.3 km) that we placed over the study area. We used a grid of 19.7×19.7 km cells, representing median male home range size for males (D. Carney, S. Courville, T. Graves, K. Kendall, R. Mace, C. Servheen, J. Waller, unpublished data). We calculated the number of individual bears detected in each grid cell, *i*, in each of the 4 hair trap and 5 bear rub sessions, *t*, and formatted the dataset as in Table 1. Within each grid cell we summarized 15 variables (Table 2) we hypothesized could influence local bear abundance and 3 variables we hypothesized could influence detection at bear rubs. We summarized landscape characteristics within ArcGIS (ESRI, Redlands, CA). Detection of bears is higher within 2 km of baited hair traps [13]. Therefore, we summarized 9 variables that plausibly could influence detection for hair traps by session in a 2 km radius around each hair trap. Our hair trap detection model component included sampling effort (# of hair traps), time (Julian day), and characteristics used in trap location selection within our 7×7 km grid cells: distance to water, road density, proximity (<500 m) of traps to a trail, amount of mesic habitat, proximity (<1 km) of traps to a recent burn, whether the trap was located in an avalanche chute, and whether the trap was on a ridge (because bears often travel along ridges). Bear rub detection covariates evaluated included sampling distribution (sd of distance among rubs in a grid cell), time (Julian days), and sampling effort (bear rub days, which we defined as the sum of the number of days since the previous visit for all bear rubs within a grid cell). In N-mixture models, detection covariates must be summarized by site, but can be constant across or vary by session, while abundance covariates must be summarized by site and cannot vary by session. We analyzed data only from grid cells sampled with both hair traps and bear rubs to ensure we were sampling the same spatial area. We repeated the analysis three times for each sex: (1) with hair traps separately (4 sessions), (2) bear rubs separately (5 sessions), and (3) combined hair trap and bear rub (9 sessions).

**Table 1:** Format of capture histories for our N-mixture model.

	Hair trap Counts			Bear Rub Counts		
Grid Cell	Session 1	…	Session 4	Session 1	…	Session 5
1	3	…	0	1	…	3
2	2	…	1	15	…	5
…	…	…	…	…	…	…
245	4	…	2	3	…	1

Each session indicates a different time period of sampling. Values are counts of individuals within a grid cell for a session.

**Table 2:** Comparison of top models from 2 datasets for grizzly bear local abundance.

Females		Weights*
Combined	Mesic Habitat, Meadow Shrub Habitat, Bear Protection Level, Historical Bear Presence, Building Density	0.90
Hair trap-only	Mesic Habitat, Meadow Shrub Habitat, Bear Protection Level, Historical Bear Presence, Building Density	0.83
Bear rub- only	Mesic Habitat, Historical Bear Presence	0.21
	Mesic Habitat, Historical Bear Presence, Area Burned 5–20 Years Ago	0.17
	Mesic Habitat, Meadow Shrub Habitat, Historical Bear Presence	0.15
Males		Weights*
Combined	Mesic Habitat, Meadow Shrub Habitat, Bear Protection Level, Historical Bear Presence	0.74
Hair trap-only	Mesic Habitat, Meadow Shrub Habitat, Bear Protection Level, Historical Bear Presence, Trail Density	0.21
	Mesic Habitat, Bear Protection Level, Historical Bear Presence, Trail Density	0.18
	Mesic Habitat, Meadow Shrub Habitat, Bear Protection Level, Historical Bear Presence	0.13
Bear rub- only	Bear Protection Level, Number Hunter Days	0.21
	Mesic Habitat, Number Hunter Days	0.19
	Precipitation, Number Hunter Days	0.14

* Weights are the proportion of MCMC samples with these covariates and represent support for models of the effect of human and habitat factors potentially influencing grizzly bear abundance in northwestern Montana, USA, in 2004. We report models up to cumulative weight = 0.5. Combined analyses include both hair trap and bear rub data.

We analyzed counts, *y_i,t_*, for each grid cell, *i*, in each session, *t*, with the N-mixture model (Supporting Information S2). The observed counts were modeled as binomial random variables, conditional on abundance, *N_i_*, but with separate detection components for each sample type using the following structure.
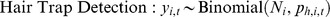


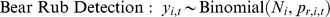
We modeled local abundance, *N_i,_* as a Poisson variable.
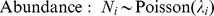
We included covariates, using a log link for the Poisson abundance process and a logit link for covariates for detection as follows:
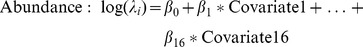


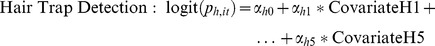


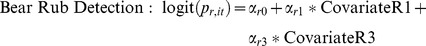
We conducted model selection using covariate indicators [14] in which the model is extended to include a collection of indicator variables, *w_j_*, having a Bernoulli distribution, that determine whether covariate *j* is included in the model (*w_j_* = 1) or not (*w_j_* = 0).
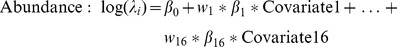
We estimated parameters using Markov chain Monte Carlo (MCMC) methods [15] with program R [16], R2WinBUGS [17], and WinBUGS [18]. If a variable is important, it should be included in the model, i.e. *w_j_* = 1, in ≥50% of the posterior samples [19], [20]. Best models are defined as variable combinations that appear most often and model weights are the percent of samples in which those variable combinations occur. After a burn-in of 10,000 samples, we saved every 20^th^ sample of 190,000 samples from the posterior distribution. We assessed model performance (mixing, convergence, and autocorrelation) visually, and with the Brooks-Gelman-Rubin statistic (BGR≤1.01) [21].

We ran the models with only the important variables to assess predictions of local abundance. For each sex, we calculated correlations among abundance estimates for hair trap-only, bear rub-only, and combined datasets. We also mapped median local abundance estimates to examine spatial differences in prediction patterns among datasets. We quantified the impact of using a single versus multiple detection method in predictions of relative local abundance by dividing by the maximum local abundance for a model, such that each model had relative abundances between 0 and 1. We mapped the grid cells where the addition of the second data source had opposite impacts on predictions, i.e. adding bear rubs to the hair trap-only analysis increased relative predicted abundance while adding hair traps to the rub-only analysis decreased relative predicted abundance.

## Results

After removing grid cells without bear rub sampling, our analysis of female detections (10.3×10.3 km grid cells) included 245 grid cells with an average of 8.51 hair traps (sd = 2.033) and 1265.5 bear rub days (sd = 1070.3, range: 28–5472 rub days, and 1–96 bear rubs) per cell (Fig. 1C). The male analysis (19.7×19.7 km grid cells) included 79 grid cells with an average of 28.5 hair traps (sd = 7.5) and 3921.7 bear rub days (sd = 3035.0, range: 70–11683 rub days, and 1–201 bear rubs) per cell (Fig. 1D).

Our first hypothesis, that the same variables would be selected as important in the hair trap-only and bear rub-only analyses, was not supported. For females, the joint hair trap and bear rub analysis identified exactly the same variables as the hair trap-only analysis, but the bear rub-only analysis only identified 2 of the 5 variables identified as important in the other analyses (Table 3). For males, the combined hair trap and bear rub analysis identified all of the variables identified in the hair trap-only analysis, except trail density, while the bear rub-only analysis identified only one unique variable (Table 3).

**Table 3:** Comparison of variable weights from 2 datasets for grizzly bear local abundance.

Sex: Scale in Km	Females:	10.3×10.3		Males:	19.7×19.7	
Data type used:	HT only	BR only	Both	HT only	BR only	Both
**Mixing of MCMC chains:**	Good	Good	Good	Slow	Good	Good
**Variables**						
Amount of Mesic Habitat	**1.00**	**0.90**	**1.00**	**0.83**	0.29	**0.92**
Bear Management Level^1^	**1.00**	0.09	**1.00**	**0.87**	0.33	**0.93**
Amount of Meadow-Shrub Habitat	**1.00**	0.32	1.00	0.49	0.03	**0.93**
Historical Presence of Bears	**0.99**	**0.97**	**1.00**	**0.99**	0.05	**1.00**
Building Density	**0.97**	0.09	**0.99**	0.02	0.03	0.02
Number Hunter-Days	0.02	0.02	0.02	0.15	**0.99**	0.10
Trail Density	0.01	0.02	0.01	**0.79**	0.03	0.02
Area Burned 5–20 Years Ago	0.01	0.37	0.01	0.01	0.01	0.01
Road Density (Total)	0.02	0.15	0.01	0.03	0.02	0.01
Avalanche Chute Area	0.01	0.01	0.01	0.17	0.03	0.01
Terrain Ruggedness	0.01	0.01	0.01	0.16	0.02	0.03
Outfitter Camp Density	0.01	0.02	0.01	0.02	0.11	0.01
Precipitation	0.02	0.04	0.01	0.04	0.20	0.01
Range of Solar Radiation	0.04	0.02	0.02	0.02	0.01	0.03
Area Burned Within 5 Years	0.02	0.01	0.01	0.05	0.02	0.02

Importance (weight) of variables influencing grizzly bear abundance in northwestern Montana, USA, in 2004. Only candidate variables for abundance, not detection, are shown. Weights for variables that were in the model ≥50% of iterations are in bold. Data include only cells with both types of sampling. HT = Hair Trap, BR = Bear Rub. See Graves et al. (In Review) for more details on specific variables. We did not include further details to maintain focus on the influence of different detection methods.

1 Experts assigned a value 1–10 to ownership categories based on efforts to protect bears including 1) attractant storage management, 2) enforcement of food storage regulations, and 3) road density and use management. Glacier National Park = 10, US Forest Service = 7, other public land = 3, and private = 1.

**Figure 2:**
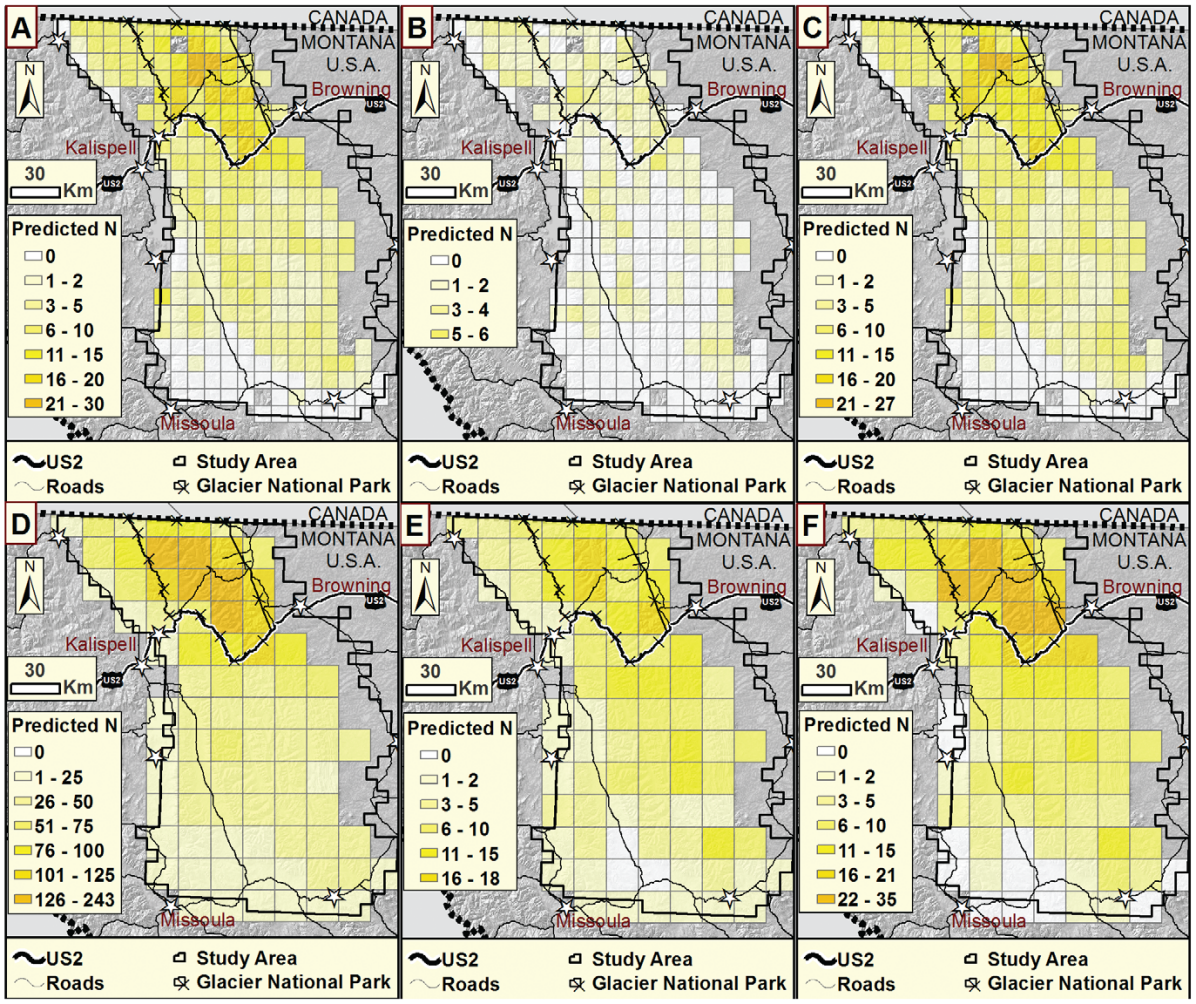
Predictions of relative local grizzly bear abundance. Predictions resulted from hair trap-only, bear rub-only, and combined models in northwestern, Montana, USA. A) Female hair trap-only. B) Female bear rub-only. C) Female combined. D) Male hair trap-only. E) Male bear rub-only. F) Male combined. Analysis included only grid cells with both detection methods.

**Figure 3:**
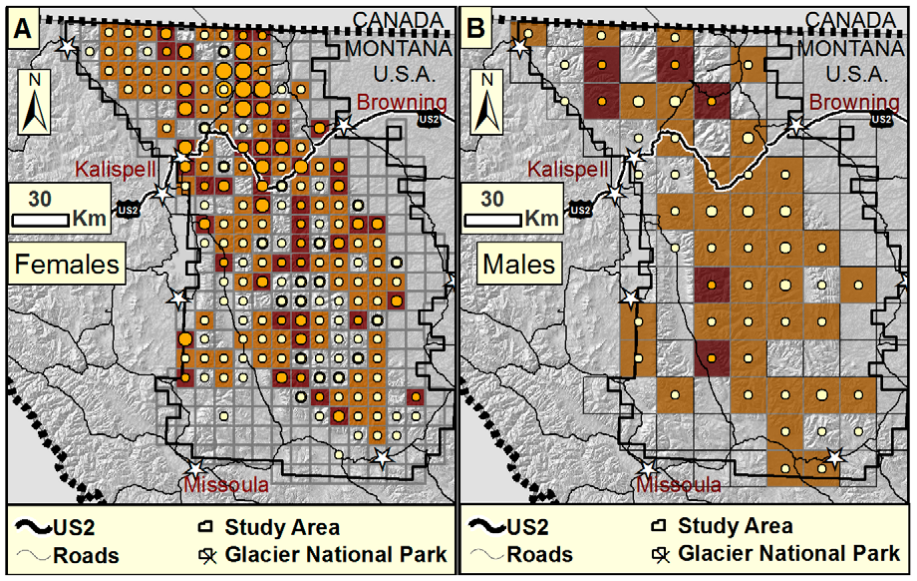
Differences in predictions of relative local grizzly bear abundance between single and multiple detection method analyses for A) Females and B) Males. Light brown cells are those where the relative abundance was predicted to increase with the addition of bear rubs and decrease with the addition of hair traps. Dark brown cells are those where the relative abundance was predicted to decrease with the addition of bear rubs and increase with the addition of hair traps. Light yellow circles indicate the degree of increase predicted by the addition of rub trees (up to 11%). Orange circles indicate the degree of increase predicted by the addition of hair traps, ranging from 0 (no circles) to 50% (large circles). Lack of circles occurs where the addition of either method decreased predictions, they stayed the same, or we did not have data from both detection methods.

Furthermore, models fitted to each of the two methods separately did not yield similar estimates of relative local abundance (Figure 2). Correlations of female local abundance estimates between hair trap-only and bear rub-only datasets were only moderate, at *r* = 0.52. Correlations of male local abundance estimates between hair trap-only and bear rub-only datasets were higher at *r* = 0.79. However, for females, estimates of local abundance for combined and hair trap-only analyses were highly correlated (*r* = 0.98), while estimates for females for combined and bear rub-only analyses were only moderately correlated (*r* = 0.66). For males both hair trap-only and bear rub-only were highly correlated with combined estimates (*r* = 0.92). Hair trap-only and combined dataset predictions of local abundance were higher within and near Glacier National Park. Predictions tended to be relatively similar for neighboring cells. Compared to hair trap-only predicted abundance, bear rub-only predictions were generally lower, with high abundance estimates distributed unevenly throughout the study area (Figure 2). A map of changes in predicted relative abundance, highlighting those grid cells where the addition of the second detection method yields increases from one detection method and decreases from the other, demonstrates the large portion of the study area where distribution predictions differ among single and multiple-detection analyses (Figure 3).

Combining both detection methods yielded higher weights for variable and model selection as predicted. The combined analyses resulted in a single model for each sex with very high weight (Table 2), while more models with lower weights resulted from single-method analyses. In particular, weights were much lower in the analyses where variables selected differed from the combined data analysis (Table 2). More models were needed to reach a combined model weight of 50% for bear rub-only analyses than in the combined data analyses for both sexes. For males, more models were also needed to reach a combined model weight of 50% for the hair trap-only analyses than in the combined analyses. Our fourth hypothesis was also supported: for variables present in both the single and combined detection methods, precision increased as expected (Supporting Information S3).

## Discussion

Our results indicate that variables identified as important were not consistent across detection methods and this influenced the distribution of relative local abundance. This suggests that our two detection methods could be sampling different segments of the population, i.e., un-modeled individual heterogeneity occurs, but because this dataset is from a natural population, and truth is unknown, alternate causes of this pattern need to be considered. Different variables could also be selected because sampling is a random process and the number of bears caught in a grid cell is stochastic. A combination of both sampling stochasticity and heterogeneity by detection methods could also explain our results. These possible explanations lead us to important and different conclusions about how to develop models from such data.

We could identify different important variables with different detection methods by chance if the detection process for one or both methods had low detection rates and high variability such that the spatial distribution of a sample varied greatly each time the population was sampled. Our median detection rates per cell per session are relatively low (female hair trap *p* = 0.07; bear rub *p* = 0.13; male hair trap *p* = 0.06; bear rub *p* = 0.13), suggesting this may occur in our data. We would also be more likely to identify different important variables by chance if high correlation existed among variables such that small, stochastic shifts in detection changed the distribution of detections to more closely match a highly correlated variable or set of variables in our multiple-variable case. This may occur in our male bear rub-only dataset. Number of hunter days was closely related to the combination of bear protection level, the amount of meadow-shrub habitat and historical bear presence (*adjusted R^2^* = 0.61), although the univariate correlation between hunter days and bear protection was low (*r* = −0.32).

If sampling variation alone caused different variables to be identified as important, inclusion of all data increases sample sizes and overall detection. We demonstrated greater support for variables selected in the combined analysis than in single detection method analyses. If the same variables were supported in the separate and joint analyses, or if there were strong reason to believe that sampling variation alone caused different variable selection, the variables identified from the combination of all available data should be reported.

However, some evidence suggests that we may have sampled different segments of the population with each method. This could result because a detection method has some un-modeled sampling bias yielding inaccurate local abundance estimates or because some bears respond differently to landscape characteristics and are more susceptible to detection by either bear rubs or hair traps (i.e., individual heterogeneity).

Bear rub sampling could have been biased in several ways. First, our bear rubs had an opportunistic distribution within the cell. We modeled this by the inclusion of detection covariates for effort and spatial distribution, but perhaps did not fully correct for variation in spatial distribution of rubs within cells. A bear's decision to use a bear rub may also depend on learned behaviors, which other bears have previously used the rub, dominance status or size of the bear rubbing, the status of other bears that used the rub, whether the bear chooses a specific path to walk along, and the amount of human trail-use, none of which are easily mapped. If human trail use decreased detection, lower counts of bears on heavily used trails could occur. Without trail use in the model, the estimates of local abundance in areas with heavily used trails would be biased low. This could create a different distribution of very local abundance across our study area and result in spurious identification of important variables. We examined the pattern of estimates of local abundance resulting from bear rub-only data, but could not identify a missing detection covariate. If covariates describing the influence of landscape on detection for a given sampling method can be developed, and at least some variation in these covariates exists, inclusion of detection covariates may correct for opportunistic sampling methods. Any abundance model could suffer from this same issue. Only the presence of different variables selected from our second detection method indicated this might have occurred.

Individual heterogeneity in detection may occur within a detection method (where 2 segments of the population exhibit different detection probabilities with that method) or between methods (if one segment of a population cannot be detected with a method). Because the sampling unit is the grid cell rather than the individual animal and the model is based on counts that do not require knowledge of individual identity, no clear way exists to incorporate individual heterogeneity within an N-mixture model. The only approach is to separately analyze groups known to differ in their detection or response to landscape. In our case, we mitigated for two forms of individual heterogeneity that likely occurred in our dataset, namely, different detection rates by sex [13], and the tendency for some (but not all) dependent cubs to be sampled along with their mothers [12]. We analyzed males and females separately, which reduced heterogeneity because male cubs would then be independent of their mother's detection and only female cub detections would be dependent.

We have confidence in hair trap-only results because the sample design is stronger, we identified the same variables in the hair trap-only and joint models, and the pattern of abundance from hair trap-only models is supported by other data [10], [12]. However, despite our efforts to mitigate for individual heterogeneity, the maximum per-cell abundance estimates from the hair trap-only analysis of males were unrealistically high (N = 150 in one cell). Other research [22] found that when detection of individuals is correlated, which could result from non-independent movement of animals, abundances were overestimated. Although it is unknown whether other forms of individual heterogeneity yield inflated abundance estimates, it seems likely that other forms of individual heterogeneity remain within our hair trap-only sample for males. Bears previously captured by humans are less likely to be detected at hair traps [12], [13]. By sampling at bear rubs we were more likely to detect this segment of the population [12]. [13] documented a male bear that was not detected although it was within 1.69 km of 17 different hair traps and speculated that preoccupation with breeding (and thus disinterest in hair traps) or other behavioral differences may lead some bears to have very low capture probabilities (∼37% of GPS-collared bears in their sample that encountered hair traps were not detected in them). Other kinds of heterogeneity in response to the landscape could also exist. For instance, adults may respond differently to the landscape than juveniles, females with cubs may respond differently than females without cubs, or bears with history of a negative interaction with humans may have stronger avoidance of human activities.

We have some support for both sampling variation and the sampling of unique segments of the population with each method. We would like to identify important variables for monitoring the population, so we conducted a thought experiment to explore the consequences of using data from each detection method. Sampling bear rubs opportunistically did not adequately sample the entire population. Hair trap-only data are based on a systematic sampling effort, and thus this analysis has stronger theoretical support. However, we want to identify variables important to abundance of all bears to focus management efforts on those variables. If some bears respond differently to the landscape and are more susceptible to detection via bear rubs, we want to know the variables important to that subset of the population. Therefore, to take a precautionary approach, we recommend that researchers in this situation report important variables for both kinds of data separately and include a discussion of strengths and weaknesses of each detection method as well as the potential for sampling multiple segments of the population. These same considerations apply to parallel situations with occupancy models.

Although we have no method to model the existence of individual heterogeneity for unknown groups within N-mixture models, the use of multiple detection methods becomes particularly important when some individuals have a zero probability of detection with one method. Indeed, it is the only way to identify that a segment of the population is not being sampled with the primary detection method and to determine the differing influence of the landscape on that segment of the population. Our comparison of results from different detection methods can identify the possibility that strong individual heterogeneity occurs, but we cannot exclude the possibility that sampling variability also plays a role. Using statistical sampling protocols should reduce the potential for spatially biased detections of individuals. We recommend against using opportunistic detection methods alone in N-mixture models for either predictions of local abundance or to identify variables affecting animal abundance. This is consistent with recommendations for occupancy studies [23].

Compared to ordinary mark-recapture and occupancy models, combining data from multiple detection methods in analysis has additional risks, namely that sampling different segments of the population that respond differently to both detection method and landscape could lead to biased estimates of the spatial distribution of local abundance. Because these models do not account for the proportion of individuals in different groups (e.g., adults versus dependent offspring), the variables are not weighted in a known way so we cannot determine whether the variables identified in the combined analysis represent the variables that are most important to the entire population. This also makes interpretation of relative predicted differences in distribution quite difficult. When researchers want to identify important variables, a combined analysis should only be used when both separate analyses identify the same variables or detection probability is extremely low. In that case, strong individual heterogeneity is unlikely to exist and combining data will improve precision of estimates for the influence of covariates. Our model assumes that detection of animals in one sampling method is independent of detection via the other sampling method, which in our case study was likely given that bears are both locally rare and largely solitary [24]. If detection methods are not independent, a variation of a robust design model could be used instead [24].

Our analysis led to some complex conclusions for incorporating multiple detection methods in N-mixture models when identification of important variables is the goal. This highlights the importance of the use of detection methods where all individuals are equally susceptible to detection, or where knowledge of the segment of the population targeted with a particular detection method is sufficient to explain results in the context of the targeted population. Our approach that 1) evaluates variables selected in separate via joint analyses, 2) assesses detection probabilities, 3) and considers correlations among variables identified as important in separate via joint analyses, will be useful for others interested in identifying variables important to local abundance.

## Supporting Information

Supporting Information S1 **Necessary permits and permissions.**
(DOC)

Supporting Information S2 **R code for models.**
(DOC)

Supporting Information S3 **Estimates of covariates in best models for areas that were sampled with both hair traps (HT) and bear rubs (BR).** These results are for a single spatial scale and do not cover the full study area.
(DOCX)
