# Supplementary material for: Balancing Precision and Risk: Should Multiple Detection Methods Be Analyzed Separately in N-Mixture Models?
Source: PLoS One. 2012 Dec 12;7(12):e49410. doi: 10.1371/journal.pone.0049410 (PMC3520967; doi:10.1371/journal.pone.0049410)
Supplement: Supporting Information S1 — Necessary permits and permissions. (DOC) [file pone.0049410.s001.doc]

Supporting Information1: Necessary permits and permissions

All hair was collected without handling of animals. We obtained permission to collect and possess bear hair non-invasively with a letter of authorization issued by the US Fish and Wildlife Service Grizzly Bear Recovery Coordinator (March 3, 2003). We obtained permission to conduct research/ access land from the National Park Service (Glacier National Park (Scientific Research and Collecting Permit #GLAC-2003-SCI-0013), Montana Department of Natural Resources (DNRC-LUL#8435), Confederated Salish and Kootenai Tribe of the Flathead Nation (letter), Plum Creek Timber Company (letter); University of Montana Forest and Conservation Experiment Station (letter); The Nature Conservancy (letter); Lincoln Electric Cooperative (letter); Mission Valley Power (letter); Missoula Electric Cooperative (letter); Sun River Electric (letter); and Glacier Electric Cooperative (letter).  Blackfeet Tribe crews collected samples on their land. We also obtained permission to conduct sampling on the land of over 100 private landowners (letters).
