# Supplementary material for: Balancing Precision and Risk: Should Multiple Detection Methods Be Analyzed Separately in N-Mixture Models?
Source: PLoS One. 2012 Dec 12;7(12):e49410. doi: 10.1371/journal.pone.0049410 (PMC3520967; doi:10.1371/journal.pone.0049410)
Supplement: Supporting Information S2 — R code for models. (DOC) [file pone.0049410.s002.doc]

Supporting Information 2

#Load packages – R2WinBUGS must already be installed on computer

library("R2WinBUGS")

#read in data file

Fdata<-read.csv("Females10_3.csv")

nrow<-dim(Fdata)[1]

#converts hair trap (HSE) and bear rub (RTE) effort covariates so that values are standardized (all variables are standardized)

HSE<-as.data.frame(Fdata[,11:14])

z<-HSE[HSE>0]

z<-as.vector(scale(z))

HSE[HSE>0]<-z

RTE<-as.data.frame(Fdata[,15:19])

z<-RTE[RTE>0]

z<-as.vector(scale(z))

RTE[RTE>0]<-z

#Create indicator variable for effort_ creates a matrix w with 0s when effort =0 and 1 otherwise

zeropR<-as.matrix(RTE)

zeropR[zeropR!=0]<-1

zeropH<-as.matrix(HSE)

zeropH[zeropH!=0]<-1

#Identify cells with both Hair traps and Bear rubs

sesssampledR<-apply(zeropR,1,sum)

sesssampledR[sesssampledR!=0]<-1

sesssampledH<-apply(zeropH,1,sum)

sesssampledH[sesssampledH!=0]<-1

bothsampled<-sesssampledH+sesssampledR

#Reduce dataset to include only cells sampled with both methods

Fdata<-Fdata[bothsampled==2,]

nrow<-dim(Fdata)[1]

Fdata2<-scale(Fdata)

#converts HSE and RTE in reduced dataset so that values are standardized (all variables are standardized)

HSE<-as.data.frame(Fdata[,11:14])

z<-HSE[HSE>0]

z<-as.vector(scale(z))

HSE[HSE>0]<-z

RTE<-as.data.frame(Fdata[,15:19])

z<-RTE[RTE>0]

z<-as.vector(scale(z))

RTE[RTE>0]<-z

#Create indicator variable for effort_ creates a matrix with 0s when effort =0 and 1 otherwise for reduced dataset

zeropR<-as.matrix(RTE)

zeropR[zeropR!=0]<-1

zeropH<-as.matrix(HSE)

zeropH[zeropH!=0]<-1

#Standardize detection covariates Julian Day (JulDayH and JulDayR)

JulDayH<- Fdata[,20:23]

J1<-(as.vector(scale(JulDayH)))

JulDayH[,1]<- J1[1:nrow]

JulDayH[,2]<- J1[(nrow+1):(nrow*2)]

JulDayH[,3]<- J1[(nrow*2 +1):(nrow*3)]

JulDayH[,4]<- J1[(nrow*3 + 1): (nrow*4)]

JulDayR<- Fdata[,24:28]

J1<-(as.vector(scale(JulDayR)))

JulDayR[,1]<- J1[1:nrow]

JulDayR[,2]<- J1[(nrow+1):(nrow*2)]

JulDayR[,3]<- J1[(nrow*2 +1):(nrow*3)]

JulDayR[,4]<- J1[(nrow*3 + 1): (nrow*4)]

JulDayR[,5]<- J1[(nrow*4+1): (nrow*5)]

#Standardize detection covariates DistH20, RdDns2K, Mesic2K

DistH20<-Fdata[,33:36]

J1<-(as.vector(scale(DistH20)))

DistH20[,1]<- J1[1:nrow]

DistH20[,2]<- J1[(nrow+1):(nrow*2)]

DistH20[,3]<- J1[(nrow*2 +1):(nrow*3)]

DistH20[,4]<- J1[(nrow*3 + 1): (nrow*4)]

RdDns2K<-Fdata[,37:40]

J1<-(as.vector(scale(RdDns2K)))

RdDns2K[,1]<- J1[1:nrow]

RdDns2K[,2]<- J1[(nrow+1):(nrow*2)]

RdDns2K[,3]<- J1[(nrow*2 +1):(nrow*3)]

RdDns2K[,4]<- J1[(nrow*3 + 1): (nrow*4)]

Mesic2K<-Fdata[,45:48]

J1<-(as.vector(scale(Mesic2K)))

Mesic2K[,1]<- J1[1:nrow]

Mesic2K[,2]<- J1[(nrow+1):(nrow*2)]

Mesic2K[,3]<- J1[(nrow*2 +1):(nrow*3)]

Mesic2K[,4]<- J1[(nrow*3 + 1): (nrow*4)]

### Write all the .txt files that Winbugs will use, including the script.txt that calls the model file (Car.txt), data files (data.txt), and initiation files (inits.txt)

cat("

model Nmix;

{

PrecBetas<-.01 #Winbugs uses precision = 1/var in specifying normal distribution so smaller is flatter

PrecIndirects<-0.01

#Priors for abundance portion of model

loglam~dflat()

beta~dnorm(0,PrecBetas)

beta2~dnorm(0,PrecBetas)

beta3~dnorm(0,PrecBetas)

beta4~dnorm(0,PrecBetas)

beta5~dnorm(0,PrecBetas)

beta6~dnorm(0,PrecBetas)

beta7~dnorm(0,PrecBetas)

beta8~dnorm(0,PrecBetas)

beta9~dnorm(0,PrecBetas)

beta10~dnorm(0,PrecBetas)

beta11~dnorm(0,PrecBetas)

beta13~dnorm(0,PrecBetas)

beta14~dnorm(0,PrecBetas)

beta15~dnorm(0,PrecBetas)

beta16~dnorm(0,PrecBetas)

#Priors for detection components of model

alphaH ~ dnorm(0,.01)

alphaR ~ dnorm(0,.01)

alpha1~dnorm(0,PrecBetas)

alpha2~dnorm(0,PrecBetas)

alpha3~dnorm(0,PrecBetas)

alpha4~dnorm(0,PrecBetas)

alpha5~dnorm(0,PrecBetas)

alpha6~dnorm(0,PrecBetas)

alpha7~dnorm(0,PrecBetas)

alpha8~dnorm(0,PrecBetas)

alpha9~dnorm(0,PrecBetas)

alpha10~dnorm(0,PrecBetas)

alpha11~dnorm(0,PrecBetas)

alpha12~dnorm(0,PrecBetas)

#priors for weights for all sub-models

w1~dbern(.5)

w2~dbern(.5)

w3~dbern(.5)

w4~dbern(.5)

w5~dbern(.5)

w6~dbern(.5)

w7~dbern(.5)

w8~dbern(.5)

w9~dbern(.5)

w10~dbern(.5)

w11~dbern(.5)

w13~dbern(.5)

w14~dbern(.5)

w15~dbern(.5)

w16~dbern(.5)

wa1~dbern(.5)

wa2~dbern(.5)

wa3~dbern(.5)

wa4~dbern(.5)

wa5~dbern(.5)

wa6~dbern(.5)

wa7~dbern(.5)

wa8~dbern(.5)

wa9~dbern(.5)

wa10~dbern(.5)

wa11~dbern(.5)

wa12~dbern(.5)

#create model

for(i in 1:R){ #loop through all grid cells

log(lam[i])<-loglam + w1*beta*Mesic[i]+ w2*beta2*Precip[i]+ w3*beta3*AvChutAr[i] + w4*beta4*MeadShrub[i]+ w5*beta5*TerRug[i]+ w6*beta6*SolRad[i]+ w7*beta7*BurnAr[i]+ w8*beta8*PercRecv[i]+ w9*beta9*BurnOv5[i]+MortRisk[i]

MortRisk[i]<- w10*beta10*TrailKm[i]+ w11*beta11*RoadDens[i] + w13*beta13*FoodStor[i] + w14*beta14*HntAll[i]+ w15*beta15*Outfits[i] + w16*beta16*Activ[i]

N[i]~dpois(lam[i])

#hair trap detection: : zeropH is 0 where no sampling occurred and thus forces detection to equal zero when effort=0

for(j in 1:4){

XH[i,j]~dbin(pH[i,j],N[i])

lpH[i,j]<- alphaH + wa1*alpha1*HSE[i,j] + wa2*alpha2*JulDayH[i,j]+ wa3*alpha3*DistH20[i,j]+ wa4*alpha4*RdDns2K[i,j] + wa5*alpha5*Ls500mTr[i,j]+ wa6*alpha6*Mesic2K[i,j]+ wa7*alpha7*InChute[i,j]+ wa8*alpha8*Ls1KmBrn[i,j]+ wa9*alpha9*Ridge[i,j] #+wa13*alpha13*Precip[i]

pH[i,j]<- (exp(lpH[i,j])/(1+exp(lpH[i,j]))) *zeropH[i,j]

}

#bear rub detection: zeropR is 0 where no sampling occurred and thus forces detection to equal zero when effort=0

for(t in 1:5){

XR[i,t]~dbin(pR[i,t],N[i])

lpR[i,t]<- alphaR + wa10*alpha10*RTE[i,t] + wa11*alpha11*JulDayR[i,t]+ wa12*alpha12*RbAvDist[i]

pR[i,t]<- (exp(lpR[i,t])/(1+exp(lpR[i,t])))*zeropR[i,t]

}

} #end loop through grid cells

} #end model description

",file="model.txt") #write model

#set up data file for WinBUGS

XH=as.matrix(Fdata[,2:5])

XR=as.matrix(Fdata[,6:10])

data <- list(XH=as.matrix(Fdata[,2:5]), XR=as.matrix(Fdata[,6:10]),

zeropR=zeropR,zeropH=zeropH, R=nrow(XH),

HSE=as.matrix(HSE), JulDayH= as.matrix(JulDayH), DistH20= as.matrix(DistH20),

RdDns2K=as.matrix(RdDns2K), Ls500mTr=as.matrix(Fdata[,41:44]), Mesic2K=as.matrix(Mesic2K),

InChute=as.matrix(Fdata[,49:52]), Ls1KmBrn=as.matrix(Fdata[,53:56]), Ridge=as.matrix(Fdata[,57:60]),

RTE=as.matrix(RTE), JulDayR= as.matrix(JulDayR),RbAvDist=as.vector(Fdata2[,30]),

Mesic=as.vector(Fdata2[,67]),Precip=as.vector(Fdata2[,66]), AvChutAr=as.vector(Fdata2[,76]),

MeadShrub=as.vector(Fdata2[,70]), TerRug=as.vector(Fdata2[,61]), SolRad=as.vector(Fdata2[,65]),

BurnAr=as.vector(Fdata2[,77]), BurnOv5=as.vector(Fdata2[,91]),PercRecv=as.vector(Fdata2[,82]),TrailKm=as.vector(Fdata2[,71]),

RoadDens=as.vector(Fdata2[,72]+Fdata2[,73]),FoodStor=as.vector(Fdata2[,68]),

HntAll=as.vector(Fdata2[,81]),Outfits=as.vector(Fdata2[,78]),Activ=as.vector(Fdata2[,64]))

Nst=apply(cbind(XH,XR),1,max,na.rm=TRUE)+1

#set up starting value files

inits <- function()

list (loglam=runif(1,.01,10), N=Nst,

beta=rnorm(1,0,1),beta2=rnorm(1,0,1), beta3=rnorm(1,0,1), beta4=rnorm(1,0,1),

beta5=rnorm(1,0,1), beta6=rnorm(1,0,1), beta7=rnorm(1,0,1), beta8=rnorm(1,0,1),

beta9=rnorm(1,0,1), beta10=rnorm(1,0,1), beta11=rnorm(1,0,1),

beta13=rnorm(1,0,1),beta14=rnorm(1,0,1),beta15=rnorm(1,0,1),beta16=rnorm(1,0,1),

alphaR=rnorm(1,0,1), alphaH=rnorm(1,0,1), alpha1=rnorm(1,0,1), alpha2=rnorm(1,0,1),

alpha3=rnorm(1,0,1), alpha4=rnorm(1,0,1), alpha5=rnorm(1,0,1), alpha6=rnorm(1,0,1),

alpha7=rnorm(1,0,1), alpha8=rnorm(1,0,1), alpha9=rnorm(1,0,1),alpha10=rnorm(1,0,1),

alpha11=rnorm(1,0,1), alpha12=rnorm(1,0,1),

w1=1,w2=1,w3=1,w4=1,w5=1,w6=1,w7=1,w8=1,w9=1, w10=1, w11=1, w13=1,w14=1,w15=1,w16=1,

wa1=1,wa2=1,wa3=1,wa4=1,wa5=1,wa6=1,wa7=1,wa8=1,wa9=1,wa10=1, wa11=1,wa12=1)

#list parameters to track

parameters <- c("N", "loglam", "alphaR", "alphaH",

"w1","w2","w3","w4","w5","w6","w7","w8","w9","w10","w11", "w13","w14","w15","w16",

"wa1","wa2","wa3","wa4","wa5","wa6","wa7","wa8","wa9","wa10","wa11","wa12")

#start WinBUGS process

out <- bugs (data, inits, parameters, "model.txt", n.thin=20,n.chains=3, n.burnin=20000, n.iter=200000,debug=TRUE,DIC=FALSE)

#get summary of results

print(out,digits=3)

##########],################

w<-(out$sims.list) #gets at just the list of variables recorded

length(w)

w2<- as.matrix(cbind(w$w1,w$w2,w$w3,w$w4,w$w5,w$w6,w$w7,w$w8,w$w9,w$w10,w$w11,w$w13,w$w14,w$w15,w$w16,w$w17,w$w18,w$w19,w$w21,w$w22,

w$wa1,w$wa2,w$wa3,w$wa4,w$wa5,w$wa6,w$wa7,w$wa8,w$wa9,w$wa10, w$wa11,w$wa12))

summary(w)

w<-w2

#concatenatews

model<-paste(w[,1],w[,2],w[,3],w[,4],w[,5],w[,6],w[,7],w[,8],w[,9],w[,10],w[,11],w[,12],w[,13],w[,14],w[,15],w[,16],sep="")

modfreq<- rev(sort(table(model)))

modwt<-modfreq/nrow(w)

modwt[1:10]

#Print best models

bestModels<-cbind(seq(1,(length(modwt)),by=1),modwt)

bestModels[1:20,]

#Print cumulative model weights

summodwt<-cumsum(modwt)

summodwt[1:9]
