# Supplementary material for: Balancing Precision and Risk: Should Multiple Detection Methods Be Analyzed Separately in N-Mixture Models?
Source: PLoS One. 2012 Dec 12;7(12):e49410. doi: 10.1371/journal.pone.0049410 (PMC3520967; doi:10.1371/journal.pone.0049410)
Supplement: Supporting Information S3 — Estimates of covariates in best models for areas that were sampled with both hair traps (HT) and bear rubs (BR). These results are for a single spatial scale and do not cover the full study area. (DOCX) [file pone.0049410.s003.docx]

Supplement 3. Estimates of covariates in best models for areas that were sampled with both hair traps (HT) and bear rubs (BR). These results are for a single spatial scale and do not cover the full study area.

| **Sex: Cell size in Km** | **Females:** | **10.3x10.3** |  | **Males:** | **19.7x19.7** |  |
| --- | --- | --- | --- | --- | --- | --- |
| **Data type used:** | **HT only** | **BR only** | **Both** | **HT only** | **BR only** | **Both** |
| **Abundance Covariates** | **Median (SD)** | | | **Median (SD)** | | |
| Intercept | 1.16 (0.90) | -0.47 (0.27) | 0.92 (0.26) | 2.75 (0.82) | 1.65 (0.30) | 1.52 (0.26) |
| Amount of Mesic Habitat | 0.36 (0.06) | 0.32 (0.08) | 0.35 (0.05) | 0.31 (0.07) |  | 0.24 (0.06) |
| Amount of Meadow-Shrub Habitat | 0.45 (0.08) |  | 0.43 (0.07) | 0.30 (0.10) |  | 0.42 (0.08) |
| Bear Management Level | 0.59 (0.08) |  | 0.50 (0.07) | 0.54 (0.09) |  | 0.51 (0.08) |
| Historical Bear Presence | 1.03 (0.29) | 1.28 (0.40) | 1.13 (0.24) | 1.01 (0.24) |  | 0.84 (0.19) |
| Building Density | 0.45 (0.11) |  | 0.41 (0.09) |  |  |  |
| Number Hunter-Days |  |  |  |  | -0.81 (0.10) |  |
| Trail Density |  |  |  | -0.35 (0.10) |  |  |
| **Detection Covariates** |  |  |  |  |  |  |
| Intercept- Hair | -1.36(1.24) |  | -2.66 (0.26) | -3.63 (0.84) |  | -2.23 (0.27) |
| Hair Trap Effort | 0.39 (0.06) |  | 0.40 (0.06) |  |  | 0.44 (0.06) |
| Julian Day | -9.00 (4.72) |  |  |  |  |  |
| Intercept- Rub |  | -0.59 (0.50) | -3.31 (0.26) |  | -2.18(0.33) | -2.19 (0.27) |
| Bear Rub Effort |  | 0.40 (0.10) |  |  | 0.41 (0.07) |  |
| Julian Day |  | -3.78 (1.35) |  |  |  |  |
| Distribution of Rubs |  |  |  |  | 0.73 (0.15) |  |
